# Temporal Patterns of Diabetes: Analyzing Disease Burden Among Adults Over 18 (2000-2021) Using the U.S. Diabetes Surveillance System (USDDS) Database

**DOI:** 10.7759/cureus.49120

**Published:** 2023-11-20

**Authors:** Onyinyechukwu B Nwachukwu, Emeka Okobi, Nwanne Onuekwusi, Ademiluyi B David, Tinuade O Adeakin-Dada, Abieyuwa B Agada, Victor C Ezeamii, Jennifer C Ezeamii, Deepali Shrivastava, Ezinne B Ezenekwe, Okelue E Okobi

**Affiliations:** 1 Neurosciences and Psychology, California Institute of Behavioral Neurosciences & Psychology, Fairfield, USA; 2 Family Medicine, American International School of Medicine, Georgetown, Guyana, USA; 3 Dentistry, Ahmadu Bello University Teaching Hospital, Zaria, Abuja, NGA; 4 Family Medicine, University of Nigeria, Nsukka, Enugu, NGA; 5 Medical Laboratory Sciences, Asokoro General Hospital, Abuja, Abuja, NGA; 6 Community and Family Medicine, Windsor University School of Medicine, Cayon, KNA; 7 Internal Medicine, College of Medicine, University of Benin, Benin, NGA; 8 Public Health, Jiann-Ping Hsu College of Public Health, Georgia Southern University, Statesboro, USA; 9 Nursing Sciences, Faculty of Health Sciences and Technology, College of Medicine, University of Nigeria, Enugu, NGA; 10 Anesthesiology, University of Minnesota, Minneapolis, USA; 11 Epidemiology and Public Health, University of Massachusetts Lowell, Lowell, USA; 12 Family Medicine, Larkin Community Hospital Palm Springs Campus, Miami, USA; 13 Family Medicine, Medficient Health Systems, Laurel, USA; 14 Family Medicine, Lakeside Medical Center, Belle Glade, USA

**Keywords:** usdds, adult aged 18 above, disease burden, diagnosed diabetes, trend analysis

## Abstract

Background

This study investigates the temporal patterns of diagnosed diabetes cases among adults aged 18 and above in the United States from 2000 to 2021, using data from the U.S. Diabetes Surveillance System (USDDS) database. The study analyzed variations in diagnosed diabetes cases based on gender, age, education, location, and race to provide insights into the changing disease burden over two decades.

Methods

A retrospective observational design was employed in analyzing data from the USDDS database. The study population comprised adults aged 18 and above with diagnosed diabetes. Descriptive statistical analysis and subgroup comparisons were performed to identify temporal trends and disparities in diagnosed diabetes cases among different demographic groups.

Results

The study uncovered significant temporal patterns in diagnosed diabetes cases among US adults. Males consistently reported higher diabetes cases (8.44%) than females (7.45%). Variations existed among age groups, with the 65-74 age group having the highest cases (19.69%) and the 18-44 age group having the lowest cases (2.34%). Disparities by race/ethnicity were evident, with non-Hispanic black individuals (11.80%) and Hispanics (11.07%) having the highest percentages, while Asians (7.84%) and whites (6.81%) had lower rates. Distinct temporal patterns emerged based on education levels, with the less than high school education group having the highest cases (11.77%), followed by those with a high school education (8.50%), and the lowest among those with higher than a high school education (6.60%).

Conclusion

The study has revealed a complex and evolving landscape of this chronic disease. Over these two decades, we observed significant fluctuations, with an overall upward trend in diagnosed diabetes cases. These findings underscore the need for a multifaceted approach to tackle diabetes effectively. Tailored interventions that consider age, gender, education, and geographic location are crucial to addressing the observed disparities in diabetes prevalence.

## Introduction

Diabetes, a chronic metabolic disorder characterized by elevated blood glucose levels, remains a significant global public health challenge with profound implications for individuals and healthcare systems. It has emerged as a pressing concern, affecting millions of people worldwide [1,2]. The prevalence of diabetes has steadily risen across the globe due to factors such as sedentary lifestyles, unhealthy diets, and an aging population [3]. According to the International Diabetes Federation, in 2021, an estimated 10.5% (536.6 million adults aged 20-79) were living with diabetes, and this number is projected to increase to 12.2% (783.2 million adults) by 2045 if current trends persist [4]. In the United States, as reported in the latest National Diabetes Statistics Report, diabetes represents a significant public health concern, with approximately 37.3 million people, or 11.3% of the population, diagnosed with the condition. Additionally, an estimated 23% (equivalent to 8.5 million people) of the total population is believed to be undiagnosed [5].

Diabetes is classified into two main types: type 1 and type 2, each distinguished by unique pathophysiological processes. In the case of type 1 diabetes, the immune system of the body mistakenly targets and eliminates the insulin-producing beta cells within the pancreas, leading to a complete shortage of insulin. In contrast, type 2 diabetes is defined by insulin resistance and relative insulin deficiency. Insulin resistance occurs when cells do not effectively respond to insulin, the hormone responsible for facilitating glucose entry into cells. Consequently, glucose remains in the bloodstream, prompting the pancreas to produce more insulin to compensate. Over time, the beta cells in the pancreas may become exhausted, leading to elevated blood glucose levels [6-8].

Study overview

The increasing prevalence of diabetes has spurred intensive research into its temporal patterns and evolving disease burden [4]. This study delves into the intricate temporal dynamics of diabetes among adults aged 18 and above in the United States, spanning the years 2000 to 2021 [9]. Utilizing the comprehensive dataset from the U.S. Diabetes Surveillance System (USDDS), this study aims to shed light on the changing prevalence and healthcare implications of diabetes over this extended period. Additionally, it examines these patterns based on gender, age, education, location, and race.

The USDDS database serves as a robust repository for a comprehensive investigation of diabetes trends, encompassing diverse data on diabetes incidence, prevalence, risk factors, management strategies, and outcomes. This dataset provides an unprecedented opportunity to understand how the landscape of diabetes has evolved over more than two decades. This examination not only provides insights into the overall disease burden but also uncovers disparities across demographic factors like gender, age, education, location, and race [9].

Understanding diabetes trends over time is crucial for predicting future burdens and planning proactive strategies. By comprehending changes in diagnosed diabetes cases, healthcare professionals and policymakers can tailor interventions, implement timely screenings, and adapt healthcare approaches. Recognizing shifts in disease burden within specific timeframes helps identify critical moments for preventive actions and resource allocation to manage potential increases in diabetes cases.

This study focuses on analyzing diabetes patterns across gender, age, education, location, and race, with significant implications for public health experts, healthcare providers, policymakers, and dedicated researchers addressing the rising diabetes crisis. By examining the USDDS database from multiple perspectives, this research aims to provide comprehensive insights to shape evidence-based interventions, guide resource allocation, and facilitate meaningful improvements in diabetes management and prevention strategies. Through a thorough exploration of temporal diabetes trends considering diverse demographic factors, our goal is to contribute to the development of well-informed public health strategies tailored to unique subpopulation needs, ultimately reducing diabetes-related health issues and fatalities.

## Materials and methods

Study design and data source

This retrospective observational study aimed to analyze temporal patterns of diabetes among adults aged 18 and above in the United States from 2000 to 2021. The primary data source for this study was the USDDS database, a comprehensive repository of diabetes-related information collected from various sources, including national surveys, health records, and clinical data. The USDDS database provided an extensive dataset enabling the examination of diagnosed diabetes cases, risk factors, and outcomes over an extended period.

Study population

The study population consisted of adults aged 18 and above residing in the United States, including individuals diagnosed with diabetes, encompassing both type 1 and type 2 diabetes. This temporal analysis spanned two decades, from 2000 to 2021, facilitating the assessment of long-term trends in diagnosed diabetes cases and the associated disease burden.

Variables of interest

The primary outcome variable was the percentage of diagnosed diabetes cases, measured as the proportion of adults within the specified age range who had received a diabetes diagnosis. The primary independent variables of interest included gender, age, education level, geographic location, and race or ethnicity. These variables allowed for a comprehensive examination of temporal patterns in diagnosed diabetes cases across diverse demographic segments.

Data analysis

Descriptive statistical analysis was conducted to characterize the temporal patterns of diagnosed diabetes cases over the study period. The overall percentage of diagnosed diabetes cases was calculated for each year within the timeframe. Temporal trends were visualized using appropriate graphical representations, such as line graphs. Subgroup analyses were performed to assess diagnosed diabetes cases based on demographic variables, including gender, age, education level, geographic location, and race or ethnicity. Within each subgroup, diagnosed case rates were calculated for each year, facilitating a detailed exploration of temporal patterns within specific demographic segments.

Ethical considerations

This study utilized de-identified secondary data from the publicly accessible USDDS database, which did not contain personally identifiable information. Consequently, ethical approval for this secondary data analysis was not required.

## Results

**Figure 1:**
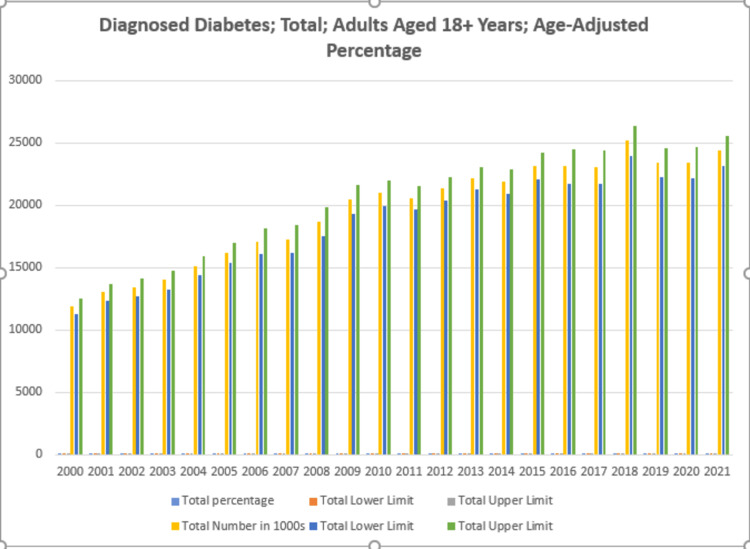
Age-adjusted percentage of diagnosed diabetes in adults aged 18+ years.

Analyzing the USDDS database from 2000 to 2021 unveiled distinct temporal patterns in diagnosed diabetes cases among adults aged 18 and above. Over the course of two decades, diabetes cases exhibited fluctuations, illustrating dynamic trends in the disease burden. A total of 429,695 cases (in thousands) were diagnosed for diabetes over the 20-year study period, with a 95% confidence interval (CI) ranging from 407,488 to 451,901. The average diagnosed diabetes case percentage was reported as 7.90% (95% CI: 7.55-8.27) during the study period (see Figure 1, Table 1).

**Table 1:** Age-adjusted percentage of diagnosed diabetes in adults aged 18+ years.

Diagnosed diabetes; total; adults aged 18+ years; age-adjusted percentage																						
Study characteristics	2000	2001	2002	2003	2004	2005	2006	2007	2008	2009	2010	2011	2012	2013	2014	2015	2016	2017	2018	2019	2020	2021
Total-percentage	6	6.4	6.5	6.6	7	7.3	7.6	7.5	7.9	8.6	8.7	8.4	8.4	8.7	8.4	8.7	8.5	8.5	9.1	8.3	8.2	8.5
Total-lower Limit	5.7	6.1	6.2	6.3	6.7	7	7.2	7.1	7.5	8.2	8.3	8.1	8.1	8.4	8	8.3	8.2	8.1	8.7	8	7.9	8.1
Total-upper Limit	6.3	6.8	6.8	6.9	7.3	7.6	8	7.9	8.4	9	9.1	8.7	8.8	9	8.7	9.1	8.9	8.9	9.6	8.7	8.6	8.8
Total-number in 1000s	11863	13006	13391	14012	15126	16186	17110	17273	18651	20490	20974	20589	21319	22173	21872	23161	23104	23048	25158	23419	23419	24351
Total-lower limit	11225	12342	12673	13265	14369	15406	16059	16167	17488	19329	19940	19682	20397	21252	20887	22080	21709	21712	23941	22247	22177	23141
Total-upper limit	12501	13670	14108	14758	15884	16966	18160	18380	19814	21652	22009	21496	22241	23093	22857	24241	24498	24385	26374	24592	24661	25561

Temporal patterns of diagnosed diabetes

**Figure 2:**
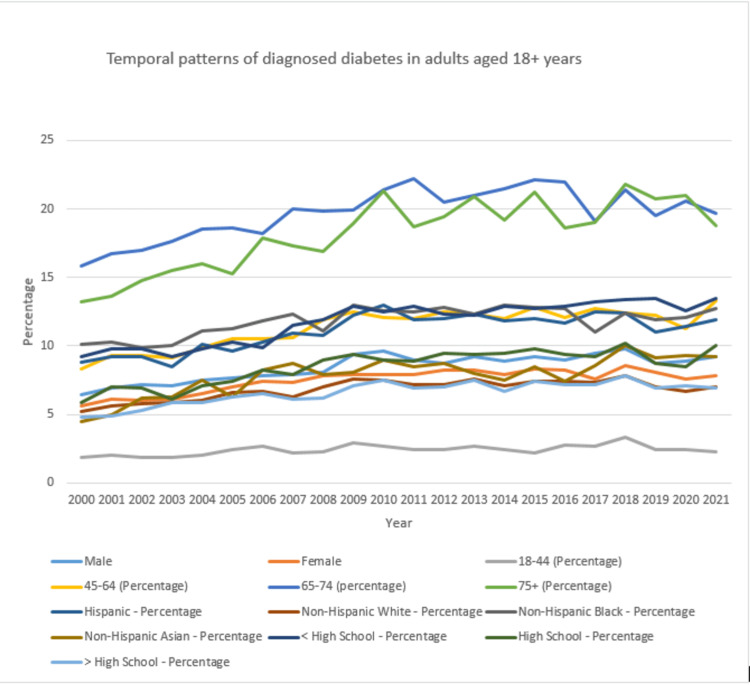
Temporal patterns of diagnosed diabetes in adults aged 18+ years.

Interactions among demographic factors, such as age, gender, education, location, and race/ethnicity, played a pivotal role in shaping the diverse temporal patterns of diagnosed diabetes cases. For instance, gender-based differences influenced diabetes case trends within specific age groups. Likewise, variations in diagnosed case percentages were observed across different education levels within specific age groups (see Table 2, Figure 2).

**Table 2:** Temporal patterns of diagnosed diabetes in adults aged 18+ years.

Diagnosed diabetes; total; adults aged 18+ years; age-adjusted percentage																							
Study characteristics		2000	2001	2002	2003	2004	2005	2006	2007	2008	2009	2010	2011	2012	2013	2014	2015	2016	2017	2018	2019	2020	2021
Gender	Male-percentage	6.4	6.9	7.2	7.1	7.5	7.7	7.8	7.9	8.1	9.4	9.6	9	8.7	9.2	8.9	9.2	9	9.5	9.8	8.7	8.9	9.2
	Female-percentage	5.6	6.1	6	6.1	6.5	7	7.4	7.3	7.8	7.9	7.9	7.9	8.2	8.2	7.9	8.3	8.2	7.6	8.6	8.1	7.6	7.8
Age data	18-44-percentage	1.9	2	1.9	1.9	2	2.4	2.7	2.2	2.3	2.9	2.7	2.4	2.4	2.7	2.4	2.2	2.8	2.7	3.3	2.4	2.4	2.3
	45-64-percentage	8.3	9.3	9.3	9.1	9.9	10.5	10.5	10.6	11.9	12.5	12.1	12	12.5	12.3	12	12.8	12.1	12.7	12.4	12.2	11.3	13.3
	65-74-percentage	15.8	16.7	17	17.6	18.5	18.6	18.2	20	19.8	19.9	21.4	22.2	20.5	21	21.5	22.1	22	19.1	21.4	19.5	20.6	19.7
	75+-percentage	13.2	13.6	14.8	15.5	16	15.3	17.9	17.3	16.9	18.9	21.3	18.7	19.4	20.9	19.2	21.2	18.6	19	21.8	20.7	21	18.8
Race	Hispanic-percentage	8.8	9.2	9.2	8.5	10.1	9.6	10.3	10.9	10.8	12.2	13	11.9	12	12.3	11.8	12	11.7	12.5	12.4	11	11.4	11.9
	Non-Hispanic White-percentage	5.2	5.6	5.8	5.9	6	6.6	6.7	6.3	7	7.6	7.5	7.2	7.2	7.6	7.1	7.4	7.4	7.3	7.8	7	6.7	7
	Non-Hispanic Black-percentage	10.1	10.3	9.9	10	11.1	11.3	11.8	12.3	11.1	13	12.6	12.5	12.8	12.3	13	12.8	12.7	11	12.4	11.9	12.1	12.7
	Non-Hispanic Asian-percentage	4.5	5	6.2	6.3	7.5	6.3	8.2	8.7	7.9	8.1	9	8.5	8.7	8	7.5	8.5	7.4	8.6	10	9.1	9.3	9.2
Education	High school-percentage	9.2	9.8	9.8	9.2	9.8	10.3	9.9	11.5	11.9	12.9	12.5	12.9	12.3	12.2	12.9	12.7	12.9	13.2	13.4	13.5	12.6	13.5
	High school-percentage	5.9	7	6.9	6.1	7.1	7.4	8.2	7.9	9	9.4	9	8.9	9.5	9.4	9.5	9.8	9.4	9.2	10.2	8.7	8.5	10
	>High school-percentage	4.8	4.9	5.3	5.9	5.9	6.3	6.5	6.1	6.2	7.1	7.5	6.9	7	7.5	6.7	7.4	7.2	7.2	7.8	6.9	7.1	6.9

Gender-based analysis

**Figure 3:**
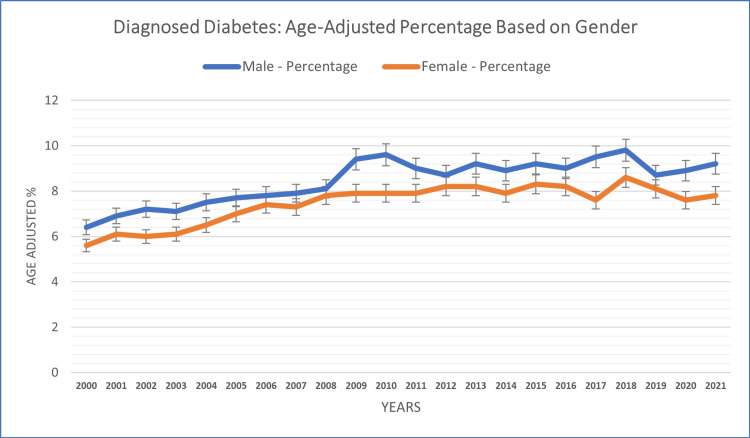
Gender-based patterns of diagnosed diabetes in adults aged 18+ years.

Gender-specific analysis revealed variations in age-adjusted diagnosed diabetes cases between males and females across the study years. Males reported a higher diagnosed diabetes case percentage (8.44%) compared to females (7.45%) throughout the study years. Among males, diagnosed diabetes cases fluctuated between 6.4% (in 2000) and 9.8% (in 2018), while among females, it ranged from 5.6% (in 2000) to 8.6% (in 2018). Diabetes cases exhibited similar trajectories for both genders, with gradual increases until around 2018 (8.6% in females, 9.8% in males), followed by slight declines until 2021 (see Figure 3).

Age-based analysis

**Figure 4:**
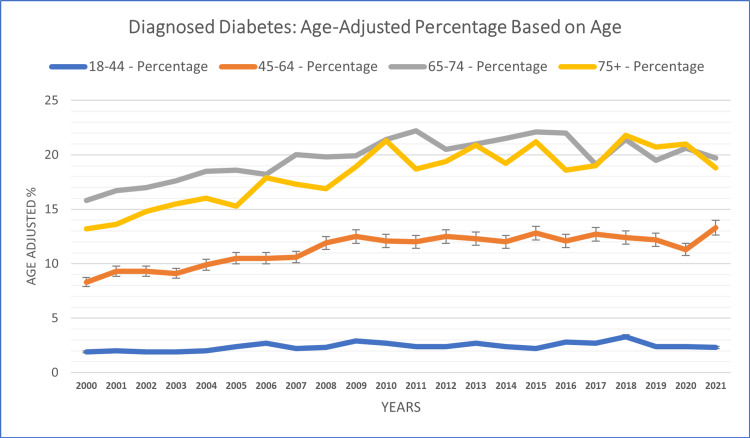
Age-based patterns of diagnosed diabetes in adults aged 18+ years.

The analysis, stratified by age groups, revealed noteworthy patterns in diagnosed diabetes cases across different life stages. Elderly individuals aged 65-74 reported the highest diagnosed diabetes case percentage (19.69%), followed by the 75+ age group (18.18%) and the 45-64 age group (11.35%). The lowest case percentage was reported in the 18-44 age group (2.34%) throughout the study years. Among adults aged 18-44, diagnosed diabetes cases ranged from 1.9% (in 2000) to 3.3% (in 2018). Similarly, for the age groups 45-64 and 65-74, fluctuations ranged from 8.3% (in 2000) to 13.3% (in 2021) and from 15.8% (in 2000) to 22.1% (in 2015), respectively. Notably, elderly individuals aged 75 and above experienced higher diagnosed diabetes cases, which fluctuated between 13.2% (in 2000) and 21.8% (in 2018) (see Figure 4).

Race/ethnicity-based analysis

Diagnosed diabetes case patterns varied significantly based on race and ethnicity. The non-Hispanic black race reported the highest diagnosed diabetes case percentage (11.80%), followed by the Hispanic population (11.07%), and the Asian racial group (7.84%). The lowest case percentage was reported in the white race (6.81%) across the study years.

**Figure 5:**
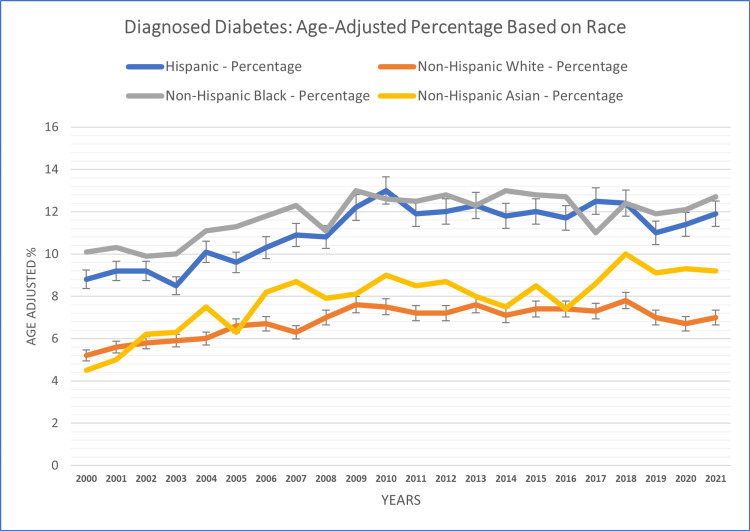
Race/ethnicity-based patterns of diagnosed diabetes in adults aged 18+ years.

White individuals experienced fluctuations ranging from 5.2% (in 2000) to 7.8% (in 2018), while Black individuals exhibited case percentages ranging from 9.9% (in 2002) to 13% (in 2014). Hispanic individuals demonstrated fluctuations between 8.5% (in 2003) and 13% (in 2010). Similarly, Asian individuals experienced percentages ranging from 4.5% (in 2000) to 10% (in 2018) (see Figure 5). The observed differences in diagnosed diabetes cases by race and ethnicity emphasize the influence of these racial and ethnic factors on the disease burden.

Education-based analysis

**Figure 6:**
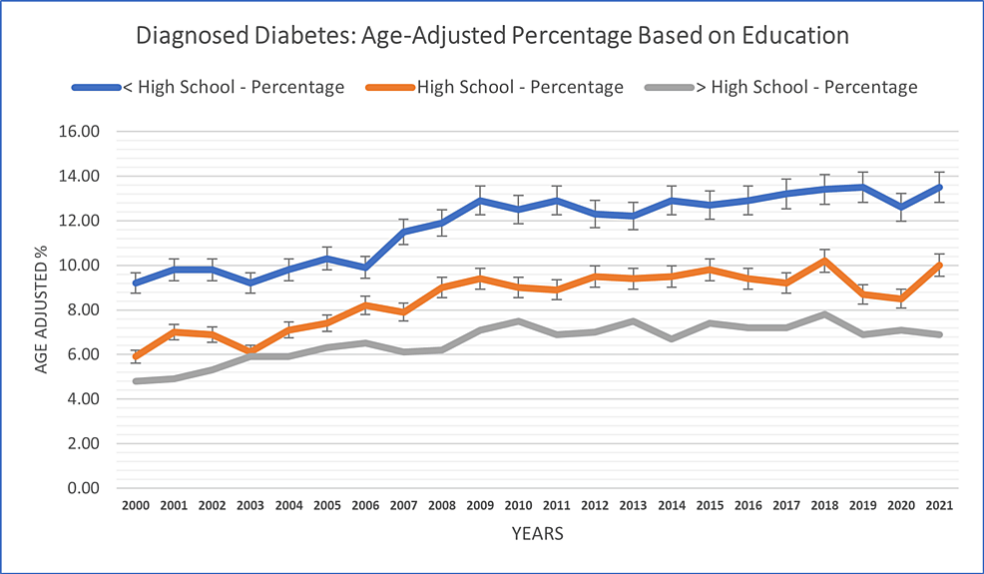
Education-based patterns of diagnosed diabetes in adults aged 18+ years.

Examining diagnosed diabetes cases by education level revealed distinct patterns among different educational strata. The population group with less than a high school education reported the highest diagnosed diabetes case percentage (11.77%), followed by the group with a high school education (8.50%), while the lowest case percentage was reported in the group with higher than a high school education (6.60%) across the study years. Adults with less than a high school education exhibited percentages ranging from 9.2% (in 2000) to 13.5% (in 2021). In contrast, those with a high school education experienced fluctuations between 5.9% (in 2000) and 10.2% (in 2018). Adults with higher than a high school education exhibited percentages ranging from 4.8% (in 2000) to 7.8% (in 2018) (see Figure 6). The differences in diagnosed diabetes cases between education levels suggest an association between educational attainment and disease burden.

Location-based analysis

**Figure 7:**
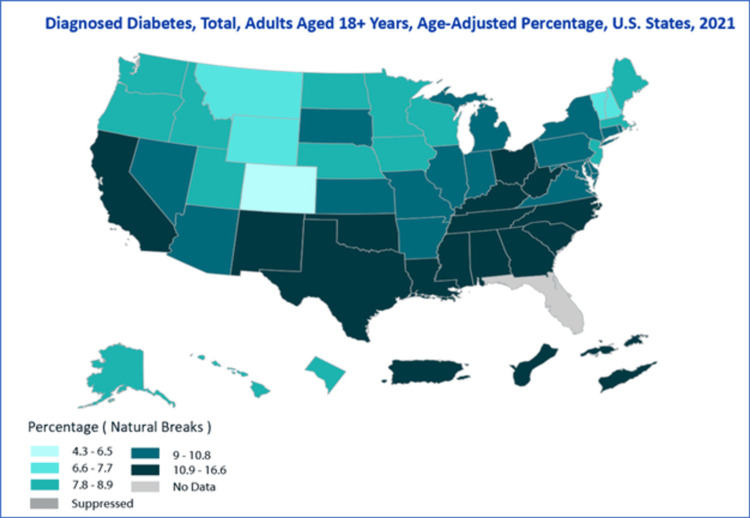
Location-based patterns of diagnosed diabetes in adults aged 18+ years.

Geographic region-based analysis highlighted disparities in diagnosed diabetes cases across different regions of the United States. The diagnosed case percentages for the top ten states are provided in Table 3. Certain states, including Mississippi, exhibited the highest diagnosed case percentage (5.5%, 95% CI: 4.0%-7.4%), followed by Alabama (5%, 95% CI: 3.6%-6.8%) and Louisiana (4.9%, 95% CI: 3.4%-6.9%). The differences in diagnosed diabetes cases by location underscored regional disparities in disease burden (see Figure 7).

**Table 3:** Location-based analysis of diagnosed diabetes in the top ten states.

State	Diagnosed diabetes case (age-adjusted percentage)	Lower limit	Upper limit
Alabama	5	3.6	6.8
California	3.8	2.9	4.9
Georgia	3.9	2.9	5.2
Kansas	3.5	3	4.2
Kentucky	4.9	3.6	6.6
Louisiana	4.9	3.4	6.9
Mississippi	5.5	4	7.4
New Mexico	4.6	3.5	5.9
Ohio	3.7	3	4.5
Pennsylvania	3.4	2.6	4.5
Rhode Island	3.5	2.4	5
South Carolina	4.6	3.6	5.8
Texas	4.6	3.6	5.9
West Virginia	4.7	3.6	6
Puerto Rico	3.4	2.3	4.9

Overall, the analysis of the USDDS database revealed complex temporal patterns in diagnosed diabetes cases among adults aged 18 and above. These patterns were characterized by variations based on gender, age, education, location, and race/ethnicity, providing valuable insights into the multifaceted nature of the diabetes burden over the past two decades.

## Discussion

The present study conducts a comprehensive examination of temporal patterns in diagnosed diabetes cases among adults aged 18 and above in the United States from 2000 to 2021, utilizing data from the USDDS database. By exploring the impact of various demographic factors, including gender, age, education, location, and race, this research offers a nuanced understanding of the evolving landscape of diabetes burden and its implications for public health interventions. Notably, this discussion includes a comparison of our findings with previous studies, enriching the context and interpretation of the observed temporal patterns.

The consistent increase in diabetes case percentages over the two-decade period, as revealed in our study, aligns with prior research findings. This upward trend underscores the urgent nature of diabetes as a global public health concern. Our gender-specific analysis consistently identifies disparities, with males reporting higher diagnosed diabetes case percentages compared to females. These findings resonate with the National Diabetes Statistics Report, which also documented substantial gender disparities in diagnosed diabetes case trends over a similar time frame [5,10]. Together, these results emphasize the importance of addressing diabetes as a shared health concern for both genders while tailoring interventions to individualized needs.

The age-specific patterns identified in our study corroborate the well-established understanding that age is a predominant factor in diabetes prevalence. As evidenced in previous research, older adults, particularly those aged 65 and above, consistently exhibit higher diabetes rates [5,10-12]. These trends align with the established association between advancing age and increased diabetes risk. Furthermore, this comparison reaffirms the significance of targeting preventive efforts toward older adults, given their elevated risk of diabetes [13].

Our study's investigation into educational disparities in diagnosed diabetes cases aligns with the findings of a study by Fang et al. in 2022 [10]. Our current analysis demonstrates a higher prevalence of diagnosed diabetes cases among individuals with lower educational attainment, while those with higher education levels exhibit comparatively lower rates. This observation is in harmony with the well-accepted concept that education acts as a determinant of health outcomes, including diagnosed diabetes cases. By corroborating previous research, our study emphasizes the enduring relevance of addressing education-related disparities in diabetes management and prevention strategies [10,14-20].

Disparities in diabetes prevalence among racial and ethnic groups are evident in our study. Non-Hispanic black individuals and Hispanics report higher diabetes case percentages compared to whites and Asians. Comparing our regional and race/ethnicity-based findings with those of Fang et al. (2022) and the National Diabetes Statistics Report (2020) shows that there are still differences in the number of people who are diagnosed with diabetes [5,10,17,21-23]. These disparities underscore the influence of social determinants of health, including access to healthcare, socioeconomic factors, and cultural differences, on diabetes prevalence. Addressing these disparities is imperative for achieving health equity. Moreover, in studying diabetes incidence rates, it is vital to highlight the importance of racial disparities in relation to obesity trends and patterns. Racial disparities help in stablishing the racial and ethnic groups that are affected by diabetes, as this will help policymakers to tailor medical interventions targeting such groups. The racial disparities in relation to diabetes prevalence are additionally important in understanding the differences in obesity risk factors, and this will help in tailoring interventions for the prevention of diabetes in affected racial groups.

Our geographic region-based analysis reveals disparities in diabetes prevalence across different regions of the United States. Certain states consistently report higher diabetes case percentages, indicating the necessity for localized public health interventions and resources to address these disparities [5,20-23].

While our study provides valuable insights into the dynamics of diagnosed diabetes cases over two decades, it is not without limitations. The accuracy of our findings relies on the quality of data within the USDDS database, which is potentially subject to inaccuracies in diagnostic coding and sampling biases. Moreover, the absence of certain variables, such as lifestyle factors and changes in healthcare practices over time, may impact the comprehensiveness of our study. Additionally, our study does not establish causality between observed trends and influencing factors. Thus, the observed limitations of the study include the various limitations related to the use of the USDDS database in the study including the limitations in the data sources. For instance, the USDDS relies on data collected from the National Diabetes Statistics Report only, which limits the amount of data used in the analysis of diabetes trends and patterns. As such, not all pertinent data is captured by the USDDS database. Consequently, the other notable limitation of the USDDS database entails its inability to track the key risk factors for diabetes, including lipids and glycemia levels, as well as its inability to capture surveillance for emergent and high-risk populations, including ethnic and racial groups. In this regard, the USDDS database only captures specific data on diabetes trends and patterns but does not capture data on the various risk factors for diabetes and the racial/ethnic groups’ diabetes prevalence rates.

Still, the other key limitation of the study includes the inability to effectively establish the cause-and-effect relationship between the observed trends and the influencing factors. In this regard, it can be noted that the causative factors of diabetes have not been included in the USDDS data, making it impossible to effectively determine the various causes and risk factors that underlie the observed trends and patterns in diabetes. Additionally, a number of potential biases that would add to the interpretation of the study outcomes with regard to their nuance and generalizability have been noted. Among such potential biases is the undercoverage bias. The data collected and used in this study might fail to accurately represent the study sample due to factors such as insufficient representation of the study population. The USDDS data does not specify the region and demographic attributes of the individuals from whom the data was collected, which makes it highly prone to biases.

Nonetheless, our study boasts several significant strengths. Its longitudinal analysis across a substantial timeframe allows for the identification of both short-term fluctuations and long-term trends in diagnosed diabetes cases. The inclusion of diverse demographic factors, including age, gender, education, location, and race, enhances the depth of our study and enables the examination of disparities across various subgroups. The utilization of the USDDS database, a nationally representative source, bolsters the credibility and generalizability of our findings. Furthermore, the comparison of our results with previous research provides valuable context and highlights consistent trends over time.

In summation, while our study is not exempt from limitations related to data accuracy, missing variables, and potential biases, its strengths lie in its longitudinal analysis, consideration of diverse demographic factors, and comparison with prior studies. These attributes collectively contribute to a more comprehensive understanding of temporal patterns in diagnosed diabetes cases among adults aged 18 and above, aiding in the formulation of informed public health strategies and interventions. It is, therefore, recommended that future studies should focus on assessing the ways in which lifestyle adjustments and healthcare procedures impact diabetes trends and patterns across different racial groups. To do this, interdisciplinary collaboration with sociologists and psychologists should be considered, as this will provide the necessary important and detailed insights into the social variables contributing to the racial disparities in diabetes incidence rates. Such an interdisciplinary approach is prone to assist in the understanding of intricate factors affecting health outcomes at much deeper levels. The approach is prone to reinforce the importance of taking on a holistic public health approach in tackling the medical, economic, and social aspects affecting diverse racial groups and contributing to the variance in diabetes incidence.

## Conclusions

In conclusion, this study enhances understanding of temporal patterns in diagnosed diabetes cases among adults aged 18 and above, considering gender, age, education, location, and race. We unveil intricate patterns that underscore the multifaceted nature of the diabetes burden. While some trends align with previous research, such as the higher prevalence of diabetes among older age groups and certain racial/ethnic disparities, our study stands out by providing a comprehensive temporal perspective. For instance, this study has disclosed a higher prevalence of diagnosed diabetes in individuals with lower educational attainment than those with higher education levels, confirming the assertion that education is a determinant of health outcomes, including diagnosed diabetes cases. Further, the study has disclosed that, with regard to gender-based disparities, males reported higher diagnosed diabetes cases than females even as older adults, including those aged 65 and above, portrayed higher diagnosed diabetes rates, confirming the established association between advancing age and increased diabetes risk. Additionally, non-Hispanic Blacks and Hispanics reported higher diabetes case percentages than whites and Asians. These variations across demographic segments highlight the need for tailored interventions addressing specific challenges faced by different groups. Importantly, these findings transcend diabetes research, informing public health policies and interventions for diabetes prevention, management, and treatment. Understanding how the diabetes burden evolves and varies across population subgroups empowers healthcare practitioners, policymakers, and researchers to craft targeted strategies that effectively address the challenges of this chronic condition.
